# A portable magneto-optical trap with prospects for atom interferometry in civil engineering

**DOI:** 10.1098/rsta.2016.0238

**Published:** 2017-06-26

**Authors:** A. Hinton, M. Perea-Ortiz, J. Winch, J. Briggs, S. Freer, D. Moustoukas, S. Powell-Gill, C. Squire, A. Lamb, C. Rammeloo, B. Stray, G. Voulazeris, L. Zhu, A. Kaushik, Y.-H. Lien, A. Niggebaum, A. Rodgers, A. Stabrawa, D. Boddice, S. R. Plant, G. W. Tuckwell, K. Bongs, N. Metje, M. Holynski

**Affiliations:** 1 School of Physics and Astronomy, Metallurgy and Materials Building, University of Birmingham, Birmingham B15 2TT, UK; 2 School of Engineering, Department of Civil Engineering, University of Birmingham, Birmingham B15 2TT, UK; 3 RSK, 18 Frogmore Road, Hemel Hempstead, Hertfordshire HP3 9RT, UK

**Keywords:** gravity, gradiometry, survey

## Abstract

The high precision and scalable technology offered by atom interferometry has the opportunity to profoundly affect gravity surveys, enabling the detection of features of either smaller size or greater depth. While such systems are already starting to enter into the commercial market, significant reductions are required in order to reach the size, weight and power of conventional devices. In this article, the potential for atom interferometry based gravimetry is assessed, suggesting that the key opportunity resides within the development of gravity gradiometry sensors to enable drastic improvements in measurement time. To push forward in realizing more compact systems, techniques have been pursued to realize a highly portable magneto-optical trap system, which represents the core package of an atom interferometry system. This can create clouds of 10^7^ atoms within a system package of 20 l and 10 kg, consuming 80 W of power.

This article is part of the themed issue ‘Quantum technology for the 21st century’.

## 1. Introduction

Since the first demonstrations over 25 years ago atom interferometry has proved to be a powerful method of precision inertial sensing. In particular, during this time cold atom-based gravity sensing has achieved the most precise measurement of local gravity to date, 6.7 pg [1]. The exceptional performance is predicted to have a tremendous impact on key topics within metrology and fundamental physics, including topics such as measurement of the fine structure constant [2], the gravitational constant ‘big G’ [3], exploration of the equivalence principle [4–6], redefinition of the kilogram as part of a watt balance [7] and numerous proposals for gravitational wave detection [8–10].

The exceptional promise of laboratory systems and their inherent low drift has led to a global push towards making transportable atom interferometry systems [11–14], targeting a range of future applications. This has focused on reductions in the size of the components needed (such as in the European project, iSense) and in the adoption of more robust technology, culminating in a range of systems being developed for outside of the typical laboratory setting. In particular, companies now offer cold atom-based gravity sensors as a commercial product targeting geophysical type applications [15,16]. These are comparable with alternative technologies for similar applications, such as falling corner cube devices [17], but are still large in size compared with the commercial gravimeters used within ground-based field surveys [18] for the detection of sub-surface structures. In particular, the typical mode of operation within micro-gravity field surveys requires sensors that can be moved by an individual and can operate within an outdoor environment for a full day, typically operating on battery power. This article examines the opportunity offered by bringing atom interferometry into gravity survey applications, in particular, highlighting the potential for drastic improvements in measurement rate and environmental robustness. This would reduce the key barriers preventing gravity surveys being used in wider applications, enabling their use within sectors such as transport or construction. However, to really bring atom interferometry into practical use a significant obstacle still resides in reducing the size sufficiently for their adoption. This article presents a significant step to overcome this barrier by discussing technology developments for the generation of a compact core cold atoms package, which in future could form the central component of an atom interferometry device.

## 2. Gravity sensing for survey applications

Gravity sensing in field surveys is used to detect features through their density contrast, and has the key advantage over alternative techniques that it is not attenuated by the intervening medium. In comparison with techniques such as ground penetrating radar, this allows the detection of features at greater depth but has the drawback that it is considerably more time consuming. Currently, there is only one gravity sensor system used in field micro-gravity surveys, the Scintrex CG-5 and the recently updated CG-6 [18], and that is used by a limited number of operators. The most obvious promise of atom interferometry based sensors arises from the potential for increased sensitivity, enabling the resolution of either smaller or deeper features than is possible with existing instruments. It has been predicted (within the Innovate UK Sigma project [19]) that a new sensor based on atom interferometry could provide enhanced detectability of targets of a factor of two in depth for a given diameter, or half the diameter at a given depth.

One example of significant impact are the many hundreds of buried mine shafts in the UK alone, whose precise position is not known, and which therefore present a hazard to the use or development of the land. Mine shafts are typically 2 m in diameter, and may be buried under several metres of spoil or made ground. Existing instruments can detect these mine shafts if they are in the upper 3–4 m. However, the majority are buried at a greater depth, and a factor of two increase in the detectability translated to being able to detect these features at depths up to 5–7 m below current ground level, which brings the majority of mine shafts that are currently undetectable into the range of detection by gravity measurements.

**Figure 1.**
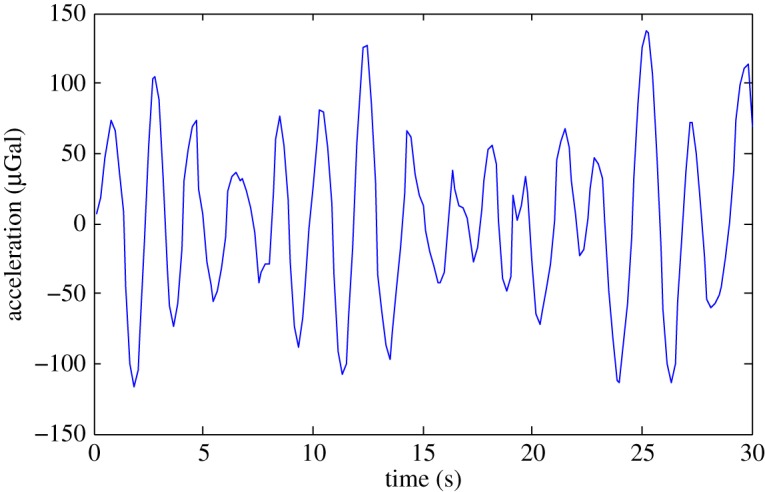
Wave noise measured by a Scintrex CG-5 gravimeter. (Online version in colour.)

The use of gravity sensing within any application is susceptible to noise arising from various sources, and understanding and accounting for these is critical for obtaining useful information from a gravity survey. In particular, gravity measurements are affected by three different types of noise sources: (i) instrument noise which varies as a function of time and the instrument used; (ii) location based noise which is dependent on the location of the measurement point and which is non-varying as a function of time and (iii) environmental noise, which is both dependent on the measurement location and variable as a function of time. In order to differentiate the signal of interest from the noise, it is important to remove the unwanted signals from the measurements. This is done by two different means: appropriate survey strategies and data post-processing. Some noise sources such as vibrations from, for example, wind noise and wave noise can be removed by sampling over a long period of time. In particular, while wind noise can be suppressed by shielding, wave noise is inherently present within the ground and occurs at sub-hertz frequencies (figure 1), rendering passive vibration isolation platforms ineffective or of impractical size. Common practice using current instruments is to average over 30 s datasets recorded three times in order to statistically reduce the noise to below the level of the signal of the target feature. This means each measuring point is occupied for at least 90 s plus the time taken to align the sensor. While longer sampling would be preferable, this becomes commercially uneconomic.

The susceptibility to noise causing a need to integrate over such long times is one of the key factors in making gravity sensing much slower than its counterpart techniques. Furthermore, this presents a significant barrier to capturing the benefits of using a more precise sensor, as while these can resolve smaller signal sizes detecting smaller signals would require even longer averaging of inherent environmental noise such as wave noise. While this may be acceptable in situations where this provided an enabling technique, such as for the detection of known targets at depth, it would require a prohibitive amount of time for exploratory surveys such as those used prior to construction.

**Figure 2.**
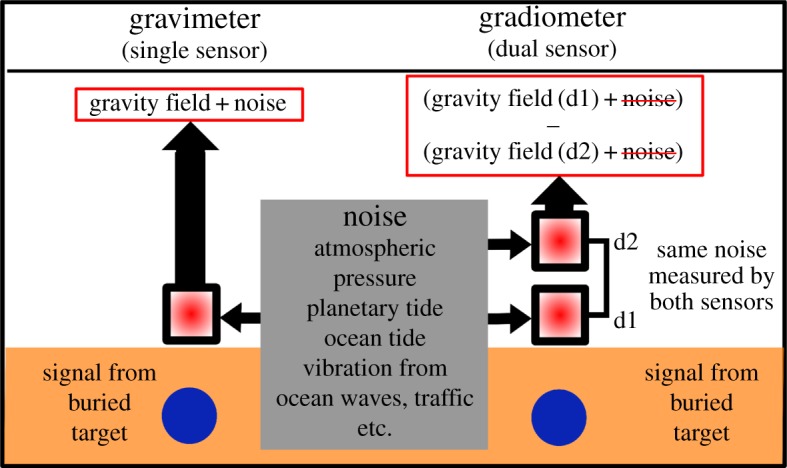
The noise cancelling effect of the gradiometer configuration in comparison to a conventional gravimeter when measuring the same buried signal. Operation of two or more gravimeters which refer to the same gravitational reference (i.e. the retroreflection mirror in an atom interferometer) necessitates common noise which is largely suppressed in the differential measurement. (Online version in colour.)

As the primary coupling of wave noise is through vertical acceleration this effect can be strongly suppressed through adoption of gravity gradient sensing. As shown in figure 2, this relies on simultaneously detecting gravity on two sensors separated by a baseline, to provide rejection of noise sources. To accurately measure, it is necessary that environmental noise sources are common mode and that both sensors are sufficiently identical in terms of drift and calibration. In contrast with two mass-on-spring based systems, atom interferometry offers the ability to directly couple two zero-drift sensors, by passing one measurement beam through both atom clouds and referencing a single mirror. This results in both atom clouds measuring the same environmental noise, and these being strongly suppressed through subtraction. In laboratory systems, for example, this has been demonstrated in [20,21] to provide better than −140 dB suppression of acceleration noise from vertical vibrations. Furthermore, this gives the added benefit of making misalignments from the vertical correlated for the pair of clouds, giving a reduced susceptibility to tilt misalignment. Table 1 summarizes how gradiometer operation via atom interferometry alters noise susceptibility compared with a conventional approach.

**Table 1.** Typical sources of noise and their approximate size in a gravity survey. The right-hand columns indicate the effect of noise on an atom interferometry (AI) based quantum sensor with arrows describing whether the effect is more or less impactful.

	source of noise	approximate scale (measured using existing gravimeter)	Will it influence an AI gravimeter?	Will it influence an AI gradiometer?
instrumental noise	tilt from vertical	0–900 μGal (depending on tilt)	✓	✓
	temperature on sensor	130 μGal mK^−1^	×	×
	linear creep on sensor springs	<2000 μGal d^−1^	×	×
environmental noise	celestial tides	up to 300 μGal in a day	✓	↓
	ocean tidal loading	≈±10 μGal	✓	↓
	atmospheric pressure	≈3–7 μGal d^−1^ (0.3 μGal hPa^−1^)	✓	↓
	seismic noise (ocean waves and earthquakes)	≈±50–300 μGal (correlated with the size and periodicity of ocean waves)	✓	×
	man-made noise (vibrations)	dependent on activity and distance from site	✓	×
	wind noise	short-term spikes of thousands of μGal depending on the weather and shelter	✓	×
	natural soil density variability	poorly understood but will depend on the type of soil	✓	↑
measurement position noise	latitude	≈0.8 μGal m^−1^ (at mid latitudes like the UK)	✓	×
	height of sensor from centre of the Earth’s gravity	308 μGal per m of elevation	✓	×
	terrain effects	≈75 μGal m^−1^ of 1800 kg m^−3^ of material but depends on size and proximity of terrain and soil density	✓	↓
	buildings	depends on size and building material	✓	↓

While alternative techniques exist for gravity gradiometry [22,23], atom interferometry has the potential to reach system sizes relevant to ground-based surveys. This offers a profound opportunity for gravity surveys, as even for devices with similar sensitivity to existing devices, suppression of tilt and wave noise would reduce alignment and integration times to seconds rather than a total of minutes—drastically reducing survey time, and allowing gravity surveys to become more prevalent. Moreover, the reduced noise manifested in the signal of interest will result in faster post-processing, even allowing on-site visualization as fewer measurement points need to be rejected.

## 3. Compact systems for atom interferometry

**Figure 3.**
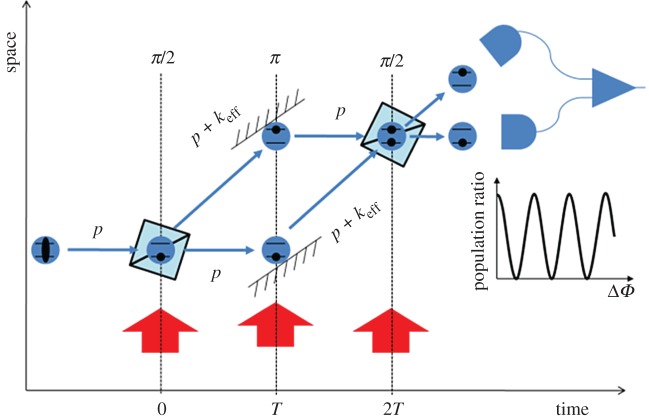
Showing the basic scheme of an atom interferometer. An atom cloud is placed in a superposition of two states through interaction with a laser beam. This gives two separated clouds travelling through space. The two are then recombined to create an interferometer. Measuring the population ratio of two states then provides a sensitive measure of gravity. (Online version in colour.)

An atom interferometer operates by dropping or throwing up a cloud of cooled atoms, which act as ideal test masses in freefall. To measure gravity, three precisely timed pulses of light are shone onto the atoms, transferring momentum to the cloud and placing the atoms into a quantum superposition of two momentum states. The first pulse is tailored to give precisely half of the atoms an extra momentum kick. This causes one half to travel more quickly through space, splitting the cloud in two. After a time *T* has passed, a second pulse is used to invert the momentum difference of the two clouds, causing them to begin to move towards each other once again. Finally, after further time *T* a third pulse is used to close the interferometer. As shown in figure 3, the result is analogous to an optical Mach–Zehnder interferometer where the roles of atoms and light have been reversed. The first and last pulses act as beam splitters, while the intermediate serves as a mirror. Rather than observing the interference pattern through optical intensity, the population of two atomic states is measured. During the sequence the atoms accumulate a phase difference due to gravity, with a sensitivity given by
3.1
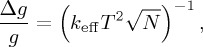
where *N* is the number of atoms probed and *k*_eff_ is the effective wavevector of the light, which determines the momentum transfer.

**Figure 4.**
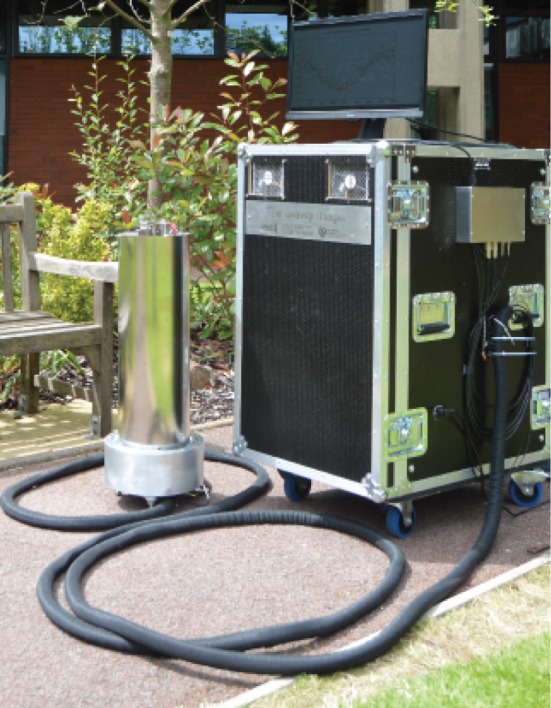
Portable gravity sensor developed at the University of Birmingham as part of the DSTL Gravity Imager programme. (Online version in colour.)

In order to assess their potential directly, a portable atom interferometry based gradiometer is being set up at the University of Birmingham. With first prototypes making their first trials outside of the laboratory, shown in figure 4, and a full gradiometry system now in construction, the system is focused on robustness and sensitivity in order to demonstrate a variety of applications. The resulting system is far more substantial than a conventional survey device, and it is clear that reaching a comparable proportion will require step changes in technology.

To address this issue, a highly portable demonstrator system for creating magneto-optical traps (MOTs) has been created. This represents a compact and ruggedized core engine, creating atom clouds which could be used as part of a measurement system. This has been used to trial existing technology options and develop novel techniques which aim at drastic reductions in size, weight and power consumption. The developed platform and techniques aim at pushing along the roadmap towards future compact and low power absolute gravity and gradiometry survey devices, informing the design and implementation of future sensor systems.

The demonstrator comprises a physics package which provides the atoms and the required environment, and a control package which provides all the laser light, stabilization and overall control.

### (a) Physics package

The physics package provides rubidium-87 atoms for cooling, and the required vacuum and electromagnetic field environment. In the compact MOT system, it comprises only an ultra-high vacuum chamber and magnetic field generation, but in a sensor would also require magnetic shielding from the external environment.

#### (i) Pyramidal magneto-optical trap

Key to the compact chamber is a pyramidal MOT design [24], in which a single input beam is split by prisms to achieve three counter-propagating beam pairs. This drastically reduces both the size of the optical delivery system and vacuum chamber, by reducing the number of inputs, and the complexity of the laser system, through not requiring the generation of individual input beams. Furthermore, the use of a single input means that real and polarization induced intensity noise are both common-mode between beam pairs. As sub-Doppler cooling techniques, ubiquitous in atom interferometry, are susceptible to variations in the beam intensity balance between counter-propagating beam pairs, this enables highly stable atom cloud temperatures and enhances the system robustness. While alternative techniques for single beam delivery exist, the pyramidal MOT is used due to its demonstrated ability to reach temperatures of order 1 μK, allowing a large portion of the prepared atoms to be probed.

#### (ii) Vacuum system

**Figure 5.**
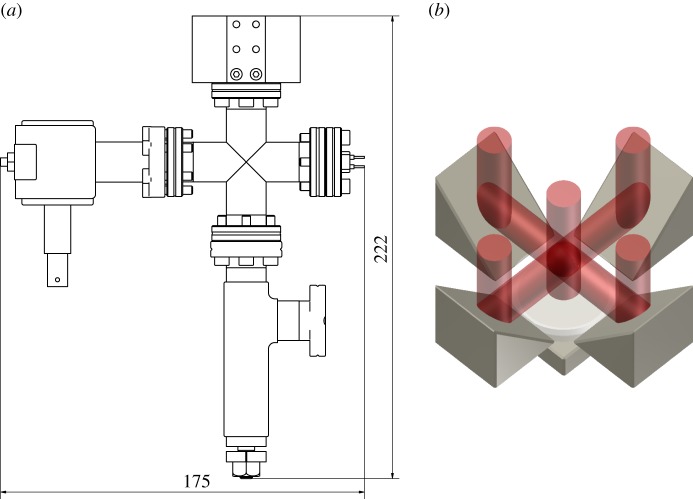
(*a*) Assembled vacuum system. The custom three-dimensional MOT region is connected to the atom source and evacuation components via a four-way cross. All commercial components are stainless steel while our chamber is manufactured in-house from titanium. (Dimensions in mm.) (*b*) Generation of the six cooling forces used in the three-dimensional MOT geometry. A single, large diameter beam is incident on four reflective prisms and a flat mirror. The prisms form four transverse cooling forces while the incident and mirror-reflected beams provide the axial forces. (Online version in colour.)

The vacuum chamber is the central component of the system around which the rest of the apparatus is mounted. The vacuum system shown in figure 5*a* comprises five components—the custom main chamber and commercial feedthrough, cross, valve and ion pump. The chamber is designed such that its radius is limited by the size of the prisms, which in turn is chosen to provide a certain atom number. The back-end of the chamber is limited by the size of the ConFlat 16 flange standard used to connect to the vacuum peripherals. Atoms are supplied through a commercial atom source.

The cylindrical chamber was machined in-house from a single piece of titanium with space at the base for the four optical prisms and a mirror and wave-plate to reflect the central portion of the beam. These combine to provide the required counter-propagating beam pairs, as shown in figure 5*b*. Each of these items was fixed in place with a small amount of vacuum adhesive [25]. The top of the chamber was machined to give a large diameter aperture and sealed with an indium glass-to-metal window to increase optical access to all of the prisms from a normally incident laser beam. Four additional window holes were machined on the side walls of the chamber to provide optical access for imaging which were also indium sealed. To provide the initial rough pumping during bakeout the chamber was connected to a turbomolecular pump, and during regular operation the system is pumped by a 2 l s^−1^ ion pump.

The ConFlat 16 standard used in the vacuum assembly currently presents the limitation for the miniaturization of the system. Use of hermetic sealing techniques, such as copper pinching which has been demonstrated at the 10^−10^ torr level [26], would allow for removal of the valve and further reduction in the package size. Another potential improvement is removal of the ion pump in favour of a completely passive pumping arrangement of getter material like those being explored in [27].

#### (iii) Magnetic fields

The MOT requires a gradient field to give spatial compression to laser cooling. The pyramidal MOT scheme used in this work employs the same magnetic field profile as experiments using three pairs of beams along orthogonal axes. This has been achieved through two approaches, initially using the typical approach of a pair of coils in a near anti-Helmholtz geometry, creating a linear gradient which is zero at the centre of the trapping region. The small size of the vacuum chamber allows compact coils to be placed close to the atoms and create gradients of 15 G cm^−1^ using coils having radius 4.5 cm, requiring a total of 6.4 W of power consumption. Although relatively low for a mains powered system, this is still considerable for a system aiming at battery operation.

**Figure 6.**
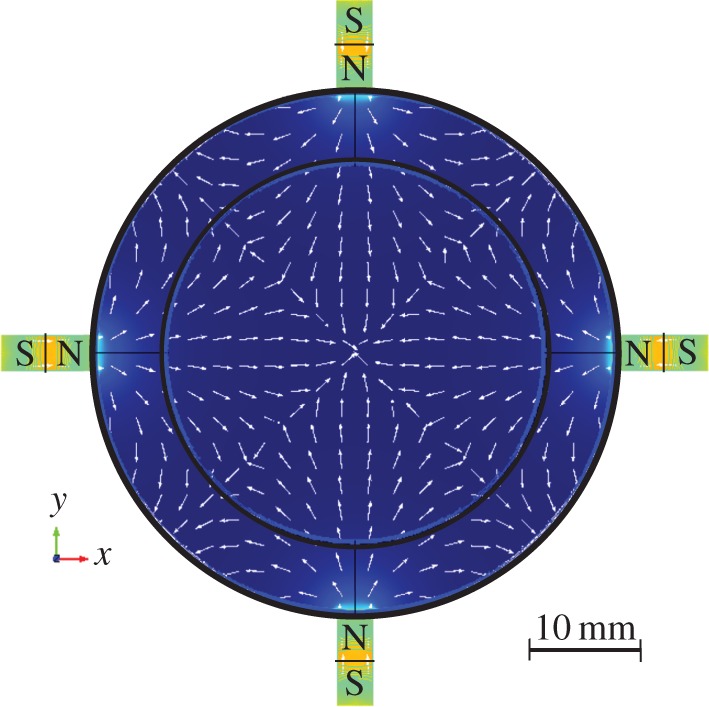
Simulated magnetic field in the *x*–*y* plane from four permanent magnets placed outside the vacuum chamber. The central region has a linear profile suitable for atom trapping. (Online version in colour.)

**Figure 7.**
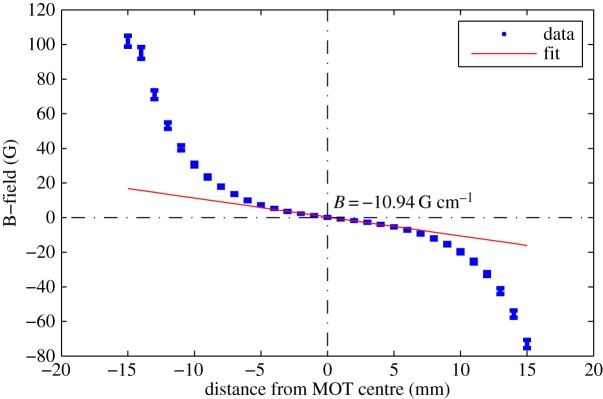
Measurement of the transverse component of the magnetic field generated by four permanent neodymium magnets in the north-inward configuration. A fitted gradient of 10.94 G cm^−1^ is within an acceptable range for rubidium trapping experiments. (Online version in colour.)

To create a more compact and low power physics package, the use of permanent magnets has been investigated. These are routinely used in cold atom experiments for atom loading, within two-dimensional MOTs and Zeeman slowers [28,29], to provide a spatially varying potential. For the generation of the fields needed to trap atoms in a three-dimensional MOT a north-inward configuration as simulated in figure 6 is employed. To realize this experimentally, four neodymium permanent magnets are mounted into a purpose built enclosure which positions the magnets precisely around the chamber. Figure 7 shows the measured gradient along the *x*-axis, with this reaching 11 G cm^−1^ at the centre. The *z*-axis gradient is twice as strong, providing a field appropriate for magneto-optical trapping. Once installed, atom clouds can be successfully realized, with no measured change in atom number or cloud distribution.

This provides a 40% reduction in radial size, limited by the size of the vacuum chamber, and almost a factor of 20 reduction in weight when compared with the coil alone. It also reduces the need for a power source, effectively removing the need for a current supply. In addition, there is a corresponding reduction of power consumption. This is of particular relevance for systems aiming at ultra-low power operation, such as space-borne or battery powered systems, but is also relevant to current systems. For example, although the compact MOT system has coils which only consume 6.4 W, the coils of a sensor system will typically consume in excess of 20 W.

However, it should be noted that employing this technique within a sensor will require further development. In particular, the inability to remove the magnetic field will inhibit reaching low temperatures using sub-Doppler cooling in the same region, requiring solutions such as a separate cooling location.

### (b) Control package

The control package provides the light generation, light frequency stabilization and required control to run a MOT system. For the current system the control is static, and would require scaling up to a timing sequence in order to operate as a sensor.

#### (i) Laser system

The light used to cool and trap the atoms is generated using mature telecom laser technology to provide a robust and compact system with narrow linewidth and high optical power [30–32]. Generation of the desired light frequencies, powers and distribution are performed within fibre and fibre integrated components to alleviate alignment issues, drastically improving resilience against effects such as mechanical shock or vibration. While this introduces concerns over polarization stability, potentially arising from mechanical and thermal drifts, this is strongly mitigated by the use of the pyramid MOT geometry which makes these common-mode to each cooling direction. The result is a system largely insensitive to external disturbance, and operable in a range of environments.

**Figure 8.**
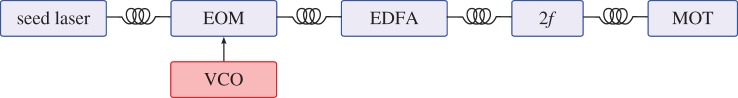
Schematic of the light generation for trapping atoms. A 1560 nm seed laser is modulated by an EOM driven by a 6.5 GHz VCO to generate frequency sidebands. After amplification by an EDFA the light is doubled in frequency via second harmonic generation in a PPLN waveguide and sent through a fibre to the experiment. (Online version in colour.)

Figure 8 shows the schematic arrangement of the laser components. A single distributed feedback fibre laser (NKT Photonics Koheras Basik) is used to seed the entire system. The two frequencies needed for cooling ^87^Rb atoms on the *D*_2_ transition are derived from the carrier and first-order frequency sideband created by phase modulation. Light from the seed laser is passed through a z-cut, lithium niobate electro-optical modulator (EOM) with a bandwidth of 10 GHz. Modulation at approximately 6.5 GHz generates a weak sideband which acts as the repumping frequency on the 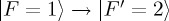 transition while the strong carrier acts as the cooling frequency on the cycling 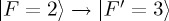 transition. The same system can be used to operate as a Raman laser system for atom interferometry by shifting the modulation frequency to 6.8 GHz and increasing the sideband power ratio accordingly.

The modulated light is amplified up to 1 W with an erbium-doped fibre amplifier (EDFA) (NKT Photonics Boostik OEM module) before being passed into a periodically poled lithium niobate (PPLN) waveguide (NTT Electronics wavelength conversion module) which achieves a conversion efficiency of >50% per watt. For typical operation the EDFA is driven at 250 mW to generate the 100 mW needed for the cooling light.

#### (ii) Laser frequency stabilization

In order to provide stabilization of the laser, we employ a technique for locking to the MOT directly, using the fluorescence signal arising from the atoms. This allows the removal of the entire atomic spectroscopy typical for magneto-optical trapping experiments, reducing overall system size and complexity, and saving optical power.

**Figure 9.**
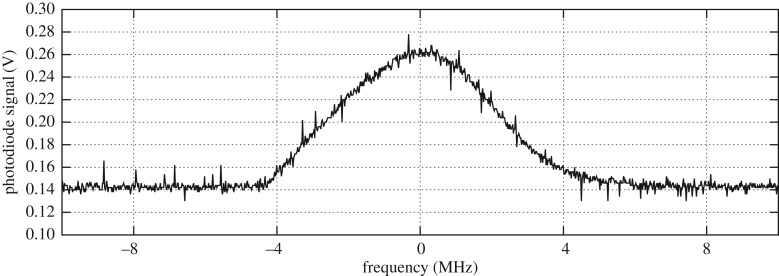
MOT signal seen by the photodiode. This plot shows the photodiode signal recorded as a function of laser frequency. The MOT is present on the 8 MHz frequency range. The signal of the MOT is approximately 46% of the total signal.

Monitoring the MOT fluorescence with a photodiode allows the creation of a locking signal (figure 9). This acts as the reference for a side-of-fringe lock by comparing a set-value against the photodiode voltage. This permits adjustment of the frequency over a range of approximately 6 MHz, centred on the MOT fluorescence maximum, and can be used for fine adjustment of the frequency.

**Figure 10.**
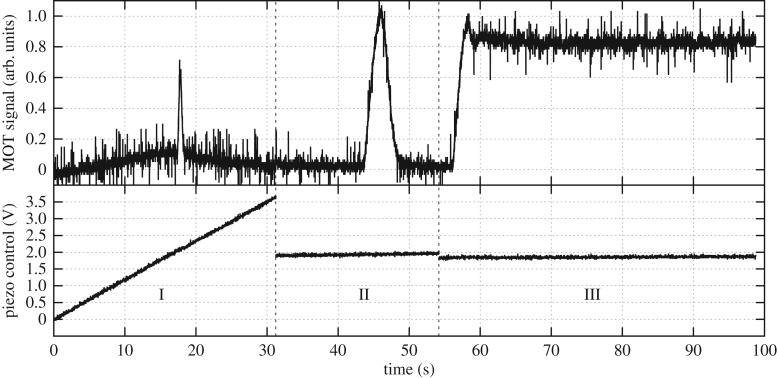
Locking stages. This plot shows the three steps used to find the MOT and stabilize the laser. Region I scans the frequency of the laser by 2.4 GHz in 31 s. Region II scans the laser on narrower frequency range of 160 MHz to determine the maximum MOT signal, set point, upper and lower bounds. Region III shows the laser frequency stabilized producing a MOT with the given set point.

In order to maintain a laser lock the control system needs a MOT present, thus requiring a multiple stage locking system. The first stage does a quick (31 s) sweep over a 2.4 GHz range to find an initial peak in the signal (region I of figure 10), this step identifies where in the frequency range the MOT is located and determines the background level.

The second stage (region II of figure 10) scans the frequency over a smaller range of 160 MHz in 21 s. The scan rate can be adjusted to account for the time scale set by the atom loading rate. This scan determines the MOT parameters required for lock control:

— maximum MOT signal = maximum measurement − background
— lower bound = 50% maximum MOT signal + background
— upper bound = 95% maximum MOT signal + background
— set point = 85% maximum MOT signal + background

**Figure 11.**
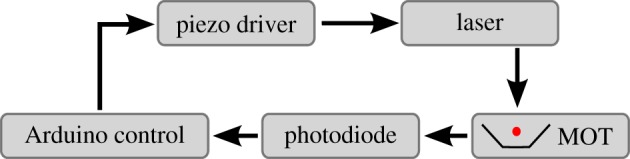
Feedback loop to control the laser. Laser light is to deliver the MOT. The MOT signal is then detected by a photodiode. The photodiode signal is fed into the Arduino control which returns a correction to the piezo driver, which in this instance changes the laser frequency. (Online version in colour.)

After the parameters for the MOT have been identified from these two stages a side-of-fringe lock is enabled. The locking scheme is shown in figure 11. The signal from the photodiode is fed into the Arduino control system, which processes the signal and feeds back into the laser piezo driver which adjusts the frequency to stabilize the laser. The program checks if the MOT signal is between the lower and upper bounds, and then either leaves the system unchanged or applies a shift to compensate. If the MOT parameters are outside acceptable bounds then a stepwise shift of 0.25 MHz is applied to the laser frequency until these conditions are met. If the parameters are acceptable then the error signal (*e*(*t*)) is calculated by the difference between the current measurement point and the predetermined set point. The correction, *Θ*(*t*), is given by

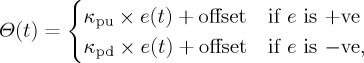
where *κ*_p_ is the proportional constant, and the offset is the position of the MOT in frequency space (region II of figure 10). The proportionality constant has two values, one to increase frequency *κ*_pu_=8 kHz and one to decrease *κ*_pd_=32 kHz as above. The need for two proportionality constants comes from the nonlinear relationship of the input voltage and frequency for the laser control. The result of this control can be seen on region III of figure 10. From this it can be seen that a lock can be achieved with this technique within 60 s of activating the laser control. To achieve a stable lock there needs to be sufficient contrast between the signal and the background, with typical values being a peak of 260 mV and a background level of 140 mV, with 46% attributed to the MOT signal.

#### (iii) System control

The control system communicates with the individual components and gathers the information. Doing this centrally allows safety features to be implemented; such as activating the seed laser first in order to prevent damage to the amplifier (EDFA). The control is implemented using a Raspberry Pi.

The Raspberry Pi was chosen due to its compact design and a large number of open source libraries. In order to facilitate the ease of communication with the Pi, a camera and touch screen produced by the same company were chosen as this can guarantee compatibility. To enhance user friendliness, all interaction with the device is done via the Raspberry Pi touch screen. The activation and locking of the laser can be done via the push of a single button, while changes to laser frequency or intensity are done by changing the value of a number. The screen also provides live data about the state of the system displaying the image of the MOT for direct monitoring while also giving information about the stability of laser temperature and frequency.

## 4. Integration

**Figure 12.**
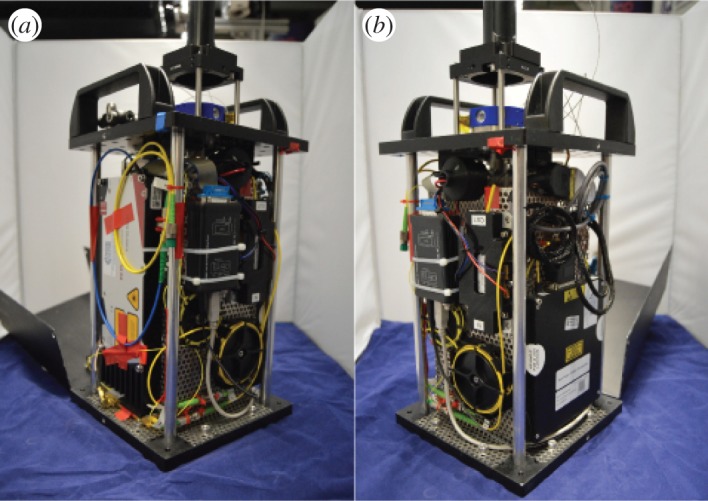
(*a*,*b*) Laser system mounted around the vacuum chamber. The entire box can be closed off and easily transported with carrying handles fixed to the top. (Online version in colour.)

The integration design focused on creating a single package with minimal overall footprint volume of the system. For this reason the vacuum assembly was designed to be tall but narrow, limited by the size of the standard CF16 valve and tee used. This is mounted onto a base plate comprised of a standard Thorlabs 20×20 cm aluminium breadboard, with a machined counterpart acting as a top plate. The electronic and laser components and the power generation are mounted around the periphery using a metallic mesh for support, as shown in figure 12. The system is sealed using a pair of metallic covers which affix onto the top and bottom plates. The optical delivery telescope is mounted onto the top plate, guiding the beam into the chamber.

Once assembled, the system has a footprint of 200×200×500 mm^3^ with the optical telescope attached, providing a significant amount of free space above the chamber. The total height of the system, minus the telescope, is 35 cm. The system weight is approximately 10 kg if using an integrated ion pump controller. If using coils to generate the magnetic field, there is free space for an additional power source to be integrated inside the package. The power consumption when running at peak power is 80 W, which is dominated by consumption within the laser system, in particular the amplifier. For a sensor system, the overall power consumption would be expected to roughly halve due to the duty cycle. The system can generate MOTs of 10^7^ atoms.

**Figure 13.**
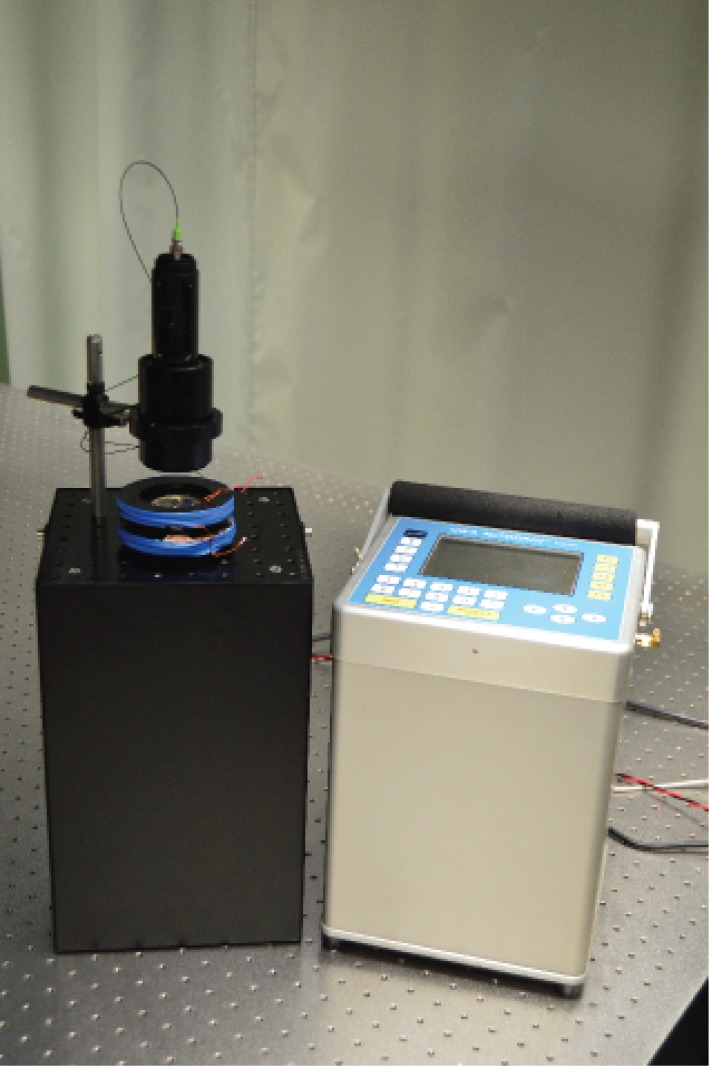
Size comparison between the compact magneto-optical trap demonstrator and the commercially available Scintrex CG-5 gravimeter. (Online version in colour.)

## 5. Conclusions

The high precision and scalable technology offered by atom interferometry has the opportunity to profoundly influence gravity surveys, in particular through the development of gradiometry systems. These are necessary in order to reduce measurement times, which is key to bringing gravity surveys into wider applications and more prevalent use. This is also important if desiring to harness future improvements in sensitivity, to prevent small signals from being obscured by environmental noise such as from waves. To push the technology envelope towards what is required in ground-based survey applications, a robust and portable atomic trap system has been developed, targeting significant reductions in size, weight and power. This forms the core package necessary for sensing, and while not operational as a sensor, it contains the majority of the required system, primarily needing extensions to the control system and magnetic shielding. While this demonstrates the feasibility of reducing the size and weight of the system to a level comparable to a conventional gravimeter, a clear barrier remains regarding power consumption. Here, while conventional sensors can operate using 5 W of power, our MOT package still requires 80 W peak power. This is still restrictive if targeting long operation times on battery power. However, it is worth noting that the predicted time saving achieved through improved measurement rates may ease requirements in this direction. Achieving drastic reductions in power consumption will require reductions in optical power generation, for example, through the development of highly stable 780 nm laser systems, to avoid efficiency losses in frequency doubling, or through techniques allowing operation with a reduced atom number.

## Data accessibility

In line with RCUK funded projects, open data will be used where applicable.

## References

[1] Dickerson SM, Hogan JM, Sugarbaker A, Johnson DMS, Kasevich MA 2013 Multiaxis inertial sensing with long-time point source atom interferometry. *Phys. Rev. Lett.* 111, 083001 (10.1103/PhysRevLett.111.083001) 24010433

[2] Wicht A, Hensley JM, Sarajlic E, Chu S 2002 A preliminary measurement of the fine structure constant based on atom interferometry. *Phys. Scr.* T102, 82 (10.1238/Physica.Topical.102a00082)

[3] Rosi G, Sorrentino F, Cacciapuoti L, Prevedelli M, Tino GM 2014 Precision measurement of the Newtonian gravitational constant using cold atoms. *Nature* 510, 518–521. (10.1038/nature13433) 24965653

[4] Schuldt T *et al* 2015 Design of a dual species atom interferometer for space. *Exp. Astron.* 39, 167–206. (10.1007/s10686-014-9433-y)

[5] Schlippert D *et al.* 2015 Ground tests of Einstein’s equivalence principle: from lab based to 10-m atomic fountains. *Proc. 50th Rencontres de Moriond ‘Gravitation: 100 years after GR’, La Thuile, Italy, 21–28 March 2015*.

[6] Bonnin A, Zahzam N, Bidel Y, Bresson A 2015 Characterization of a simultaneous dual-species atom interferometer for a quantum test of the weak equivalence principle. *Phys. Rev. A* 92, 023626 (10.1103/PhysRevA.92.023626)

[7] Merlet S, Kopaev A, Diament M, Geneves G, Landragin A, Pereira Dos Santos F 2008 Micro-gravity investigations for the LNE watt balance project. *Metrologia* 45, 265–274. (10.1088/0026-1394/45/3/002)

[8] Graham PW, Hogan JM, Kasevich MA, Rajendran S 2013 New method for gravitational wave detection with atomic sensors. *Phys. Rev. Lett.* 110, 171102 (10.1103/PhysRevLett.110.171102) 23679702

[9] Graham PW, Hogan JM, Kasevich MA, Rajendran S 2016 A resonant mode for gravitational wave detectors based on atom interferometry. *Phys. Rev. D* 94, 104022 (10.1103/PhysRevD.94.104022)

[10] Chaibi W, Geiger R, Canuel B, Bertoldi A, Landragin A, Bouyer P 2016 Low frequency gravitational wave detection with ground based atom interferometer arrays. *Phys. Rev. D* 93, 021101(R) (10.1103/PhysRevD.93.021101)

[11] Wu B, Wang Z, Cheng B, Wang Q, Xu A, Lin Q 2014 The investigation of a *μ*Gal-level cold atom gravimeter for field applications. *Metrologia* 51, 452–458. (10.1088/0026-1394/51/5/452)

[12] Hauth M, Freier C, Schkolnik V, Senger A, Schmidt M, Peters A 2013 First gravity measurements using the mobile atom interferometer GAIN. *Appl. Phys. B* 113, 49–55. (10.1007/s00340-013-5413-6)

[13] Bongs K *et al* 2014 isense: A technology platform for cold atom based quantum technologies. In *Research in optical sciences*, p. QTu3B.1. Washington, DC: Optical Society of America.

[14] Ménoret V, Geiger R, Stern G, Zahzam N, Battelier B, Bresson A, Landragin A, Bouyer P 2011 Dual-wavelength laser source for onboard atom interferometry. *Opt. Lett.* 36, 4128–4130. (10.1364/OL.36.004128) 22048340

[15] http://aosense.com/ (accessed 9 December 2016).

[16] http://www.muquans.com/ (accessed 9 December 2016).

[17] http://www.microglacoste.com/fg5.php (accessed 9 December 2016).

[18] http://www.scintrexltd.com/gravity.html (accessed 9 December 2016).

[19] Studies of industrial gravity measurement applications. http://www.rsksigma.co.uk/ (accessed 9 December 2016).

[20] Snadden M, McGuirk J, Bouyer P, Haritos K, Kasevich M 1998 Measurement of the Earth’s gravity gradient with an atom interferometer-based gravity gradiometer. *Phys. Rev. Lett.* 81, 971–974. (10.1103/PhysRevLett.81.971)

[21] McGuirk JM, Foster GT, Fixler JB, Snadden MJ, Kasevich MA 2002 Sensitive absolute-gravity gradiometry using atom interferometry. *Phys. Rev. A* 65, 033608 (10.1103/PhysRevA.65.033608)

[22] http://www.lockheedmartin.co.uk/us/products/gravity-gradiometry.html (accessed 9 December 2016).

[23] Lee JB 2001 Falcon gravity gradiometer technology. *Explor. Geophys.* 32, 247–250. (10.1071/EG01247)

[24] Lee KI, Kim JA, Noh HR, Jhe W 1996 Single-beam atom trap in a pyramidal and conical hollow mirror. *Opt. Lett.* 21, 1177–1179. (10.1364/OL.21.001177) 19876291

[25] Agilent. 2014 Agilent Torr Seal low vapor pressure resin sealant, datasheet.

[26] Du S *et al* 2004 Atom-chip Bose-Einstein condensation in a portable vacuum cell. *Phys. Rev. A* 70, 053606 (10.1103/PhysRevA.70.053606)

[27] Rushton JA, Aldous M, Himsworth MD 2014 Contributed review: the feasibility of a fully miniaturized magneto-optical trap for portable ultracold quantum technology. *Rev. Sci. Instrum.* 85, 121501 (10.1063/1.4904066) 25554265

[28] Tiecke TG, Gensemer SD, Ludewig A, Walraven JTM 2009 High-flux two-dimensional magneto-optical-trap source for cold lithium atoms. *Phys. Rev. A* 80, 013409 (10.1103/PhysRevA.80.013409)

[29] Cheiney P *et al.* 2011 A Zeeman slower design with permanent magnets in a Halbach configuration. *Rev. Sci. Instrum.* 82, 063115 (10.1063/1.3600897) 21721682

[30] Peil S, Crane S, Ekstrom CR 2003 High-efficiency frequency doubling for the production of 780 nm light. In *IEEE Int. Frequency Control Symp. and PDA Exhibition Jointly with the 17th European Frequency and Time Forum, Tampa, FL, USA, 4–8 May 2003*, pp. 159–161. Piscataway, NJ: IEEE (10.1109/FREQ.2003.1275080)

[31] Carraz O, Lienhart F, Charrière R, Cadoret M, Zahzam N, Bidel Y, Bresson A 2009 Compact and robust laser system for onboard atom interferometry. *Appl. Phys. B* 97, 405–411. (10.1007/s00340-009-3675-9)

[32] Sané SS, Bennetts S, Debs JE, Kuhn CCN, McDonald GD, Altin PA, Close JD, Robins NP 2012 11 W narrow linewidth laser source at 780 nm for laser cooling and manipulation of rubidium. *Opt. Express* 20, 8915–8919. (10.1364/OE.20.008915) 22513602

